# Trait mindfulness and competitive-race flow in swimmers: the mediating role of attentional control

**DOI:** 10.3389/fpsyg.2026.1938433

**Published:** 2026-10-05

**Authors:** Lipeng Luo, Biwei Xu

**Affiliations:** 1 College of Outdoor Sports, Guilin Tourism University, Guilin, Guangxi, China; 2 College of Physical Education and Health, Guangxi Normal University, Guilin, Guangxi, China

**Keywords:** attentional control, competitive swimming, mediation analysis, pacing, race flow, trait mindfulness

## Abstract

**Background and purpose:**

Mindfulness training benefits athletes’ psychological states and performance, but how mindfulness translates into competitive experience remains unclear. This study examines whether attentional control is the psychological pathway from trait mindfulness to race flow in competitive swimmers, and whether this pathway extends to objective pacing accuracy.

**Methods:**

A prospective, multi-wave observational design measured trait mindfulness (T1), attentional control (T1/T2), and race flow (post-race) at separate time points. The main analysis included 280 adult swimmers who had trained for over 3 years and whose main event included the 400 m freestyle. Near-race attentional control served as the mediator and baseline attentional control as a covariate; competitive anxiety and level were controlled. A mediation model with cluster-robust standard errors (clustered by team) was applied, and the indirect effect was estimated with 5,000 bias-corrected bootstrap samples. Pacing accuracy was an exploratory downstream outcome.

**Results:**

Attentional control accounted for the association between trait mindfulness and race flow (indirect effect 0.127, 95% CI [0.071, 0.192]; mediated proportion 43.4%), with a significant direct effect, indicating partial mediation; the attentional control–flow path was the strongest link. At the between-person level, race flow did not predict late-race pacing deviation, and no flow dimension was significant after correction. The reverse mediation test was also significant, limiting interpretation to temporal-order inference.

**Conclusion:**

The findings characterise attentional control as a psychological pathway linking trait mindfulness to race flow, but this study did not find sufficient evidence that the pathway extends to objective pacing. The study was not preregistered and is not a clinical trial.

## 1 Introduction

The application of mindfulness training in competitive sport has built a fairly solid evidence base. Systematic reviews and meta-analyses show that mindfulness-based interventions can improve several psychological indicators in athletes and have a positive effect on athletic performance (Wang et al., 2023), and broader evidence also confirms the overall effectiveness of mindfulness interventions for health and psychological functioning (Zhang et al., 2021). As the related literature has grown rapidly over recent decades (Baminiwatta and Solangaarachchi, 2021), researchers’ attention is shifting from whether mindfulness works to how it works. However, existing work still describes this psychological mechanism inadequately. Many studies use self-report scales or competitive results as outcomes, but few examine the intermediate psychological processes through which mindfulness is translated into a specific competitive experience, and questions at the mechanistic level remain to be refined (Goldberg et al., 2022). Events such as competitive swimming, which rely heavily on attentional regulation, provide a suitable setting in which to test this mechanism under real competition. This study therefore focuses on one specific question: whether attentional control is the psychological pathway through which trait mindfulness is translated into race flow.

Placing attentional control as the intermediate link in this pathway has a theoretical basis. Neuroimaging and cognitive studies indicate that mindfulness is closely related to attention networks and neurocognitive control systems, and that its effects are largely achieved through attentional and self-regulatory mechanisms (Weder, 2022). At the trait level, a stable and long-term mindful disposition means that a person is better able to notice and allocate attentional resources, and is therefore more likely to maintain effective attentional control in demanding situations. In other words, trait mindfulness provides a relatively stable psychological foundation for attentional control, which forms the first link that this study aims to test.

The link from attentional control to state flow is the most critical part of the theoretical chain and the one that most needs to be argued clearly. Flow is a mental state marked by task engagement, highly focused attention, and reduced self-monitoring, and neuroscientific evidence links it to attentional focus and the corresponding neural regulatory systems (van der Linden et al., 2021). Entering and maintaining this state is not passive relaxation but requires continuous attentional regulation. Flow is better understood as an adaptive state of engagement in which challenge matches skill and arousal is moderate (Scheepers and Keller, 2022), rather than as mere relaxation. People with higher attentional control should therefore find it easier to form and sustain flow during high-pressure competition. It should be noted that attentional focus is not equivalent to flow: excessive, rigid hyperfocus differs from the smooth, effortless quality of flow (Ashinoff and Abu-Akel, 2021), and this distinction suggests that attentional control benefits flow through flexible rather than extreme attentional allocation. At the same time, although flow is often divided into nine dimensions, recent research emphasises treating it as a single, unified core experience rather than as nine fully independent processes (Norsworthy et al., 2021; Norsworthy et al., 2023a,b). Given the marked heterogeneity in how flow is defined and measured (Peifer et al., 2022), this study uses the total score of the state flow scale to capture this core experience and treats dimension-level analyses as exploratory.

After the psychological chain of mindfulness–attentional control–race flow has been established, a natural follow-up question is whether the chain extends further to objective competitive performance, that is, to late-race pacing accuracy. Here the study does not assume a positive extension; whether the chain extends to objective pacing is treated as an open, exploratory question. Accurate pacing depends on continuous, explicit monitoring of speed and effort, whereas the reduced self-monitoring and immersion that accompany flow may be in tension with such controlled monitoring; moreover, high attentional focus is not equivalent to optimal performance regulation (Ashinoff and Abu-Akel, 2021). More broadly, the relationship between psychological variables and objective athletic performance is inherently complex and often shows small or unstable effects (Lochbaum et al., 2022); although sport flow can be influenced by psychological training, the evidence for its effect on objective performance is likewise inconsistent (Goddard et al., 2023). On this basis, the study sets the question of whether flow extends to objective pacing as an exploratory test and expects it to be non-significant at the level of stable individual differences.

Taken together, this study proposes the following hypotheses within a multi-wave prospective observational design in competitive swimmers. H1: trait mindfulness positively predicts race flow (total effect). H2 (core): attentional control mediates H1, so that trait mindfulness acts on race flow through attentional control. H3 (exploratory): the psychological chain extends to late-race pacing accuracy and, at the between-person level, is expected to be non-significant. It should be noted that, although trait mindfulness, attentional control, and race flow are measured at separate waves to support the hypothesised direction, the observational design and measurement timing are not sufficient to establish causality; the inference for H2 is therefore limited strictly to temporal-order inference rather than a causal claim. The nine-dimension analysis of the FSS-2 is declared in advance as exploratory and is not set as a numbered hypothesis. Restricting participants to competitive athletes also helps to characterise the relationships among attention, anxiety, self-confidence, and flow within the same competitive context (Domínguez-González et al., 2024), and thus to test more accurately the conditions under which the proposed psychological pathway holds.

## 2 Materials and methods

### 2.1 Design and ethics

This study used a prospective, multi-wave observational design with no random assignment and no intervention; data were collected only at predetermined waves in natural training and competition settings. The independent variable, the mediator, and the outcome were separated in time: trait mindfulness and baseline attentional control were collected at T1, near-race attentional control at T2, and race flow and split pacing immediately after each race. This temporal structure was a design premise for later interpreting the mediation direction as temporal-order inference, not causal evidence. The analysis plan (the decision rule for the timing of the attentional-control mediator, the rule for excluding the first pacing split, and the exploratory directional predictions for the nine dimensions) was written into the ethics application before data collection but was not publicly preregistered; the independent verifiability of confirmatory inference was therefore limited, and the related conclusions are all stated in exploratory or temporal terms. The study assigned no health-related intervention, does not meet the World Health Organization definition of a clinical trial, and was not registered as one. All participants were adults and provided written informed consent; individual data were not fed back to coaches, so as to avoid affecting selection and to reduce social-desirability pressure (Kegelaers et al., 2022). The study protocol was approved by the Ethics Committee of Guilin Tourism University (approval number GTU-2025-261).

### 2.2 Participants

Participants were adult competitive swimmers who had trained systematically for at least 3 years, whose main event included the 400 m freestyle, and who completed three standardised test races. Restricting the sample to competitive athletes kept the three psychological variables and performance within the same competitive context (Wang et al., 2023; Domínguez-González et al., 2024). Participant-level exclusions targeted factors that might confound the interpretation of trait mindfulness: a history of regular meditation and previous systematic psychological-skills training. Race-level exclusions applied only to the exploratory pacing analysis and included in-race coaching instructions, an effort rating below 8, injury or illness, and timing anomalies. The sample flow was as follows: 355 recruited, 342 completed T1, 310 completed T2, 280 in the main analysis, and 219 in the sensitivity sample. For exploratory pacing, 168 of an initial pooled set of 766 races were excluded under preset rules, leaving 598 valid races from 249 participants. The sample size was determined by a Monte Carlo simulation targeting the indirect effect, and the minimum detectable effect size was reported for three sample levels.

### 2.3 Procedure

The schedule of the three waves and the measures collected at each are shown in Table 1. T1 collected trait mindfulness (FFMQ), a parallel mindfulness index (MIS), baseline attentional control (ACS_T1), social desirability (MCSDS-13), background information, and informed consent. T2 (median interval 39 days) collected near-race attentional control (ACS_T2). Each participant then took part in three standardised 400 m test races (long-course pool, electronic timing); competitive state anxiety (CSAI-2R), training load, and sleep were collected before each race, and race flow (FSS-2) and split pacing were collected immediately after each race. Because pre-race state variables may affect both psychological experience and performance, they were included as control variables (Květon et al., 2021).

**Table 1:** Study waves and measurement schedule.

Wave	Time	Measures	Variable role
T1	Baseline	FFMQ, MIS, ACS_T1, MCSDS-13, background, informed consent	Independent variable X; covariates
T2	Median 39 days later	ACS_T2	Mediator M
Races ×3 (pre-race)	Before each race	CSAI-2R, training load, sleep	Covariates
Races ×3 (post-race)	After each race	FSS-2, split pacing	Outcome Y; exploratory downstream

The wave structure, in which the three variables were measured at non-overlapping time points, is shown in Figure 1 and provides the design basis for the temporal-order inference of H2.

**Figure 1:**
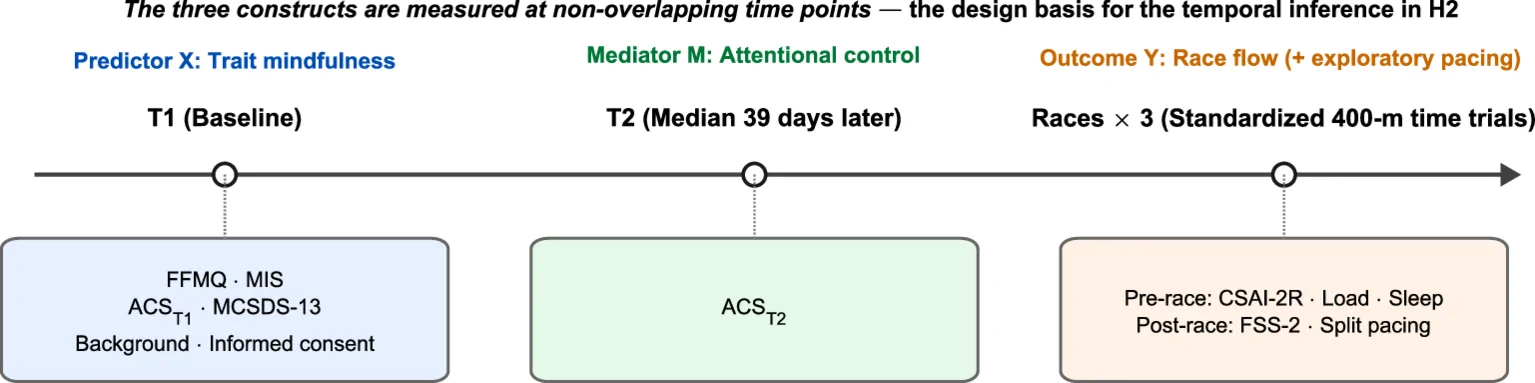
Study flow and multi-wave measurement timeline.

### 2.4 Measures

#### 2.4.1 Trait mindfulness

Trait mindfulness was measured with the Five Facet Mindfulness Questionnaire (FFMQ; 39 items, α = 0.93), and a parallel self-report of mindfulness (MIS; α = 0.80) was collected as an auxiliary measure. The multidimensional FFMQ was used rather than a unidimensional scale, and trait mindfulness, state mindfulness, and mindfulness-practice experience were strictly distinguished (Baminiwatta and Solangaarachchi, 2021).

#### 2.4.2 Attentional control

Attentional control was measured with the Attentional Control Scale (ACS). The test–retest correlation between the baseline (ACS_T1) and near-race (ACS_T2) administrations was *r* = 0.351 (*p* < 0.001); the shared variance was limited, suggesting that the measure contained a substantial occasion-specific component. Accordingly, the near-race ACS_T2 served as the mediator M and ACS_T1 as a covariate, so that near-race attentional control could be examined while baseline level was controlled. The relatively low test–retest coefficient indicates a substantial occasion-specific component, so ACS_T2 is best read as near-race attentional control rather than a stable trait; this is addressed in the Discussion.

#### 2.4.3 Race flow

Race flow was measured with the long form of the state flow scale (FSS-2; 36 items, α = 0.95), with the total score as the main outcome. Treating flow as an overall construct captured by its core experience, and using the total score as the main outcome, is consistent with recent integrative arguments (Norsworthy et al., 2021); the nine dimensions were declared in advance as exploratory and were not set as numbered hypotheses. Because flow instruments vary greatly in version and scoring, this study explicitly reports the version and scoring used (Peifer et al., 2022; Norsworthy et al., 2023b), and the immediate post-race self-report is consistent with treating flow as a measurable psychological state (Norsworthy et al., 2023a).

#### 2.4.4 Exploratory downstream outcome

The exploratory downstream outcome was objective pacing deviation. The relative deviation from target for each split was computed from the second split onward; the first split was excluded because of systematic contamination from the dive start. The late-race index Δ_late was the mean deviation of the seventh and eighth splits, with positive values indicating slower than target and negative values faster than target. Using objective pacing as an exploratory performance outcome reflects the idea that performance can be characterised jointly by self-report and objective indicators (Lochbaum et al., 2022).

#### 2.4.5 Covariates

Covariates were ACS_T1, CSAI-2R (*α* = 0.83), competitive-level points, sex, age, years of systematic training, team, and social desirability (MCSDS-13); the last also served as a partial control for common-method bias.

### 2.5 Data preprocessing and missing data

Scale scores were computed as item-level person-means: when a participant had a small number of missing items on a scale, the mean of that participant’s remaining items on the same scale was used, and no multiple imputation or full-information maximum likelihood was applied. The main analysis was based on a participant-level complete-case sample, defined as participants with valid scores on the three core variables (FFMQ, ACS_T2, and FSS-2), yielding *N* = 280. Natural attrition at each wave was reported transparently in the sample flow (355 → 342 → 310 → 280 → 219), and under this design the analysis introduced no additional imputation assumptions. The data had team-level clustering (14 teams), so all regressions used cluster-robust standard errors clustered by team. The exploratory pacing data were cleaned under race-level rules after the first split was removed, and races with timing anomalies were excluded only in the exploratory analysis. The main-analysis sample and the pacing sample (598 races, 249 participants) were two separate sets and were reported separately, not mixed.

### 2.6 Statistical analysis

#### 2.6.1 Measurement model

Each scale was scored as the item-level person-mean (as described in Section 2.5) and used as an observed indicator; no latent-variable confirmatory factor model was built, so CFI, TLI, RMSEA, and SRMR are not reported. Reliability is reported as Cronbach’s α (FFMQ 0.93, ACS_T2 0.91, FSS-2 0.95, CSAI-2R 0.83, MIS 0.80, MCSDS-13 0.81). Discriminant validity relied mainly on HTMT, which was below 0.85 for all construct pairs. For convergent validity, the AVE based on a principal-component approximation was below 0.37 for all constructs, short of the conventional 0.50 threshold. AVE is, however, a conservative index: as Fornell and Larcker (1981) note, a construct can retain adequate convergent validity even when AVE falls below 0.50, provided its composite reliability is high, and internal consistency was high for every scale in this sample (α = 0.80–0.95). Moreover, a principal-component approximation does not separate common from unique variance and therefore tends to inflate rather than deflate loadings, so the reported values are, if anything, an upper bound; the shortfall is thus treated as a genuine but conservative result rather than as an artefact of the approximation. Convergent validity accordingly relied mainly on the established construct validity and high internal consistency of each scale, with AVE reported transparently rather than as the sole criterion, a limitation addressed in the Discussion. Multicollinearity was assessed with variance inflation factors, all below 2.3. Common-method bias was mitigated by separating the three variables in measurement time and by including social desirability as a covariate, rather than by Harman’s single-factor test. Model diagnostics and collinearity checks were performed with the corresponding statistical tools (Lüdecke et al., 2021).

#### 2.6.2 Core mediation model

The core mediation model used trait mindfulness as the independent variable, ACS_T2 as the mediator, and the FSS-2 total score as the outcome, controlling for ACS_T1, CSAI-2R, and competitive-level points. Path coefficients are reported in standardised form; standard errors were estimated with cluster-robust methods clustered by team, and confidence intervals for the indirect effect were estimated with 5,000 bias-corrected bootstrap samples. Because only 14 teams were available, the indirect effect was additionally checked with a Rademacher wild cluster residual bootstrap at the team level with 9,999 resamples. The model specification and covariate entry are shown in Figure 2; because the design was observational, the mediation direction was treated only as temporal-order inference and not as a causal claim (van Lissa, 2022).

**Figure 2:**
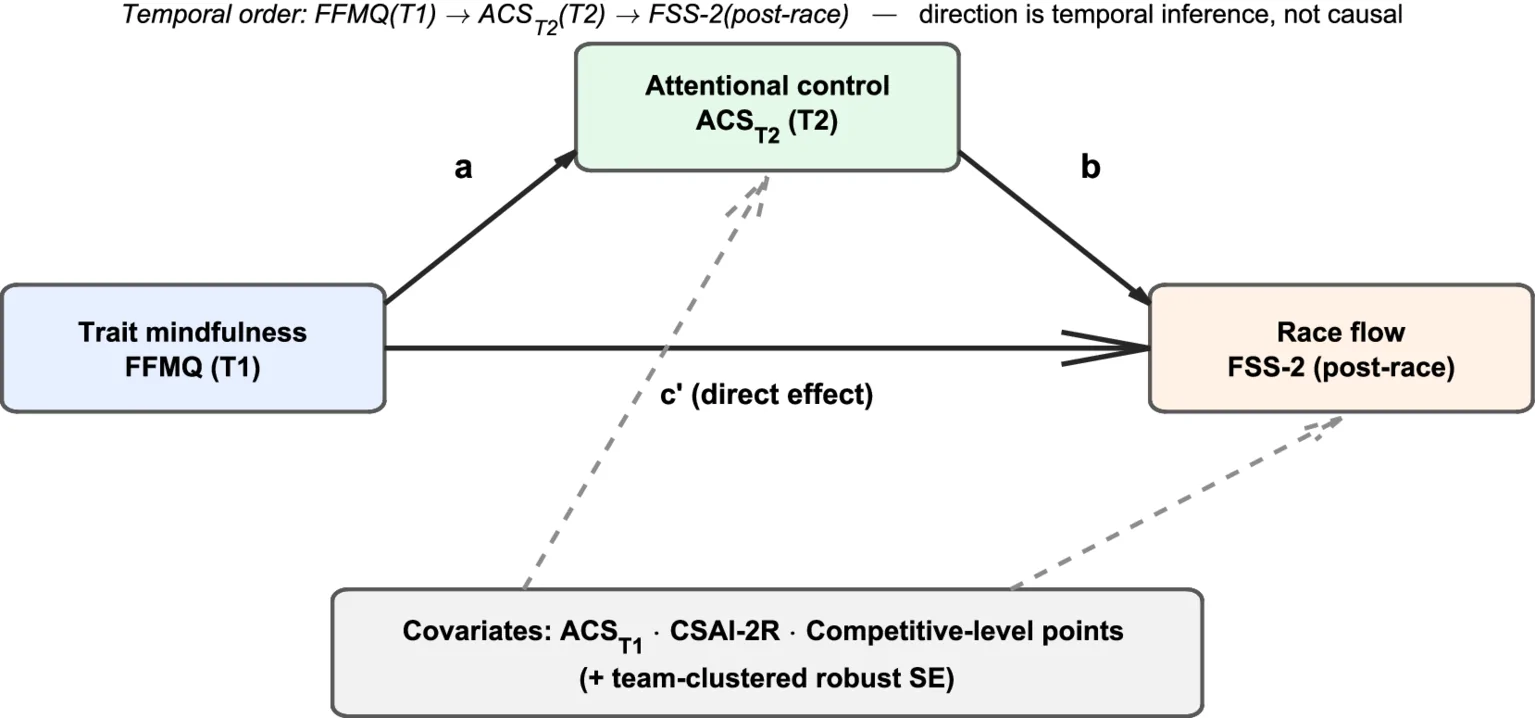
Path specification and covariate structure of the attentional-control mediation model.

#### 2.6.3 Whether the chain extends to pacing

Whether the chain extended to pacing was an exploratory analysis, in which the relationship of the FSS-2 to Δ_late was decomposed into three levels: race, within-person, and between-person. Under the preset framework, the main conclusion was fixed at the between-person level; signals at the within-person level served only as evidence of within-person covariation, not as evidence of transmission through individual differences. The flow–pacing association was always reported with its level labelled: at the participant level in the main sample (*N* = 280) the zero-order r was −0.267, and at the between-person level in the cleaned pacing sample (*n* = 249) the zero-order r was −0.129 and β was −0.099 after covariates were included. The effects of the nine dimensions on pacing are reported after Holm correction.

#### 2.6.4 Robustness checks

Robustness checks included reverse mediation, participant-level exclusion (*N* = 219), and extended covariates, with sex-stratified analysis as an option. Reverse mediation swapped the outcome and the mediator (trait mindfulness → flow → attentional control) and was expected to be equally significant; precisely because the reverse path also held, the text explicitly acknowledges that the observational design and measurement timing were not sufficient to establish causal direction, and the causal interpretation of the mediation result was limited to temporal-order inference.

## 3 Results

### 3.1 Participants, descriptive statistics, and measurement model

The sample flow was 355 recruited, 342 completing T1, 310 completing T2, 280 entering the main analysis, and 219 in the sensitivity sample after participant-level exclusion; the exploratory pacing analysis was based on 598 valid races from 249 participants after cleaning. Attrition at each wave was reported transparently in the sample flow, and the main analysis included 280 participants with post-race flow data and computable scale scores.

The internal consistency of all scales was good: FFMQ α = 0.93, ACS_T2 α = 0.91, FSS-2 α = 0.95, CSAI-2R α = 0.83, MIS α = 0.80, and MCSDS-13 α = 0.81. Discriminant validity relied mainly on HTMT, which was below 0.85 for all three core construct pairs (FFMQ–ACS_T2 0.478, FFMQ–FSS-2 0.395, ACS_T2–FSS-2 0.615). The AVE based on a principal-component approximation was below 0.37 for all constructs, not reaching the 0.50 threshold; convergent validity therefore relied mainly on the established construct validity of each scale and the high internal consistency in this sample, a limitation addressed in the Discussion. All variance inflation factors were below 2.3 (the highest being 2.26 for years of systematic training), so there was no substantial collinearity. The test–retest correlation between ACS_T1 and ACS_T2 was *r* = 0.351 (*p* < 0.001), supporting the use of the near-race ACS_T2 as the mediator and ACS_T1 as a covariate. The 280 participants were distributed across the 14 teams with a median of 20.5 participants per team (range 13–28). The intraclass correlation coefficient for race flow from a random-intercept null model was approximately zero (the team-level variance component was estimated at the boundary), indicating negligible team-level clustering of the primary outcome, so the cluster-robust standard errors represent a conservative specification rather than a correction for substantial dependence; the design effect implied an effective sample size of approximately 280, and the wild cluster bootstrap interval for the indirect effect was consistent with the bias-corrected interval reported in Section 3.2. Descriptive statistics, the correlation matrix of the main variables, and reliabilities are shown in Table 2.

**Table 2:** Descriptive statistics, correlation matrix, and reliabilities (*N* = 280).

Variable	M (SD)	1	2	3	4	5	6	7	8
1 FFMQ	3.03 (0.55)	(0.93)							
2 MIS	3.00 (0.57)	0.520	(0.80)						
3 ACS_T1	2.47 (0.61)	0.290	0.156	(0.91)					
4 ACS_T2	2.43 (0.61)	0.364	0.209	0.348	(0.91)				
5 FSS-2	3.05 (0.48)	0.383	0.170	0.266	0.574	(0.95)			
6 CSAI-2R	2.49 (0.33)	−0.230	−0.084	−0.062	−0.187	−0.220	(0.83)		
7 Competitive-level points	602.3 (124.5)	0.140	0.129	0.140	0.048	0.118	0.089	—	
8 Δ_late	3.16 (1.69)	−0.228	−0.134	−0.080	−0.251	−0.267	0.097	−0.202	—

Values in parentheses on the diagonal are Cronbach’s α. The correlation between FSS-2 and Δ_late, *r* = −0.267, is the zero-order correlation at the participant level in the main sample (*N* = 280); in the cleaned pacing sample (*n* = 249) the between-person zero-order correlation was *r* = −0.129 (see Section 3.3). AVE (principal-component approximation): FFMQ 0.273, MIS 0.269, ACS_T1 0.358, ACS_T2 0.361, CSAI-2R 0.274, FSS-2 0.369. HTMT and VIF are given in the text.

### 3.2 Main mediation: mindfulness → attentional control → flow

After ACS_T1, CSAI-2R, and competitive-level points were controlled, trait mindfulness positively predicted attentional control (a = 0.264, *p* = 0.003), and attentional control positively predicted race flow (b = 0.480, *p* < 0.001); the latter was the strongest link in the chain. The total effect of trait mindfulness on flow was significant (c = 0.292, *p* < 0.001), and the direct effect remained significant after the mediator was included (c′ = 0.165, *p* = 0.008), indicating partial mediation. The indirect effect a × b was 0.127, with a 95% CI of [0.071, 0.192] based on 5,000 bias-corrected bootstrap samples, which excluded 0. As a small-cluster check, the Rademacher wild cluster residual bootstrap at the team level (9,999 resamples) gave a consistent 95% CI of [0.063, 0.192]. Thus, H2 was supported; the mediated proportion was 43.4%. Path coefficients and effect estimates are shown in Table 3, and the standardised path diagram is shown in Figure 3. Because the design was observational, these directions are temporal-order inferences and not causal claims.

**Table 3:** Path coefficients and indirect effect of the mediation model (*N* = 280).

Path / effect	Standardised estimate	95% CI	*p*
a Mindfulness → Attentional control	0.264	[0.106, 0.422]	0.003
b Attentional control → Flow	0.480	[0.389, 0.572]	<0.001
c′ Mindfulness → Flow (direct)	0.165	[0.050, 0.281]	0.008
c Mindfulness → Flow (total)	0.292	[0.160, 0.424]	<0.001
Indirect effect a × b	0.127	[0.071, 0.192]	—

Mediated proportion 43.4%, partial mediation; the CI of the indirect effect excludes 0. The primary CI for the indirect effect is from 5,000 bias-corrected bootstrap samples; the Rademacher wild cluster residual bootstrap CI was [0.063, 0.192]. SEs are cluster-robust, clustered by team.

**Figure 3:**
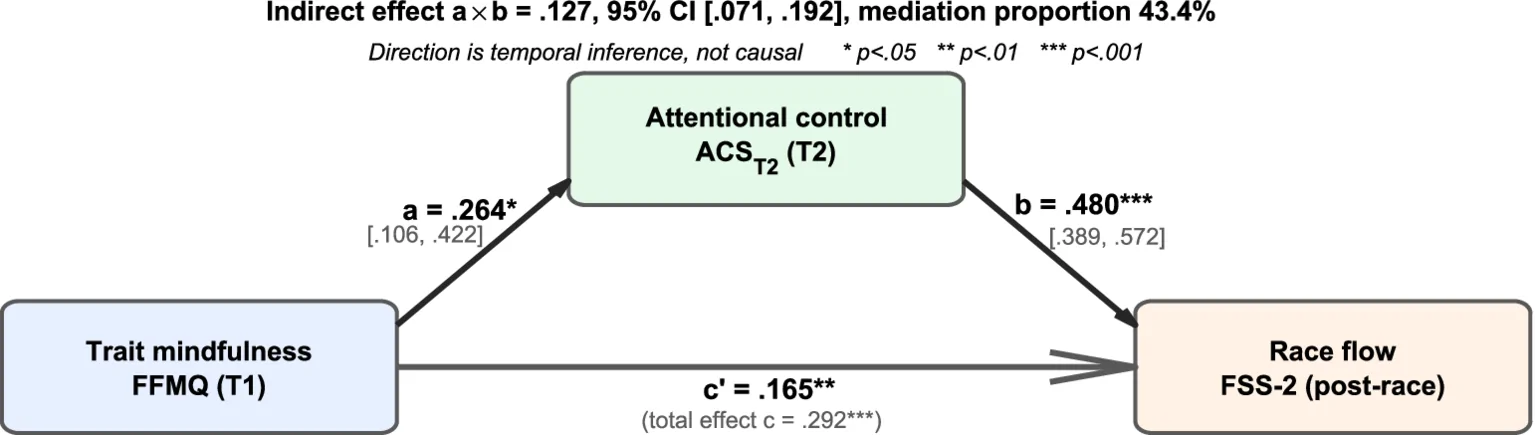
Mediation path diagram of trait mindfulness predicting race flow via attentional control (standardised coefficients with 95% confidence intervals).

### 3.3 Exploratory analysis: no sufficient evidence that the pathway extends to objective pacing

After the relationship of the FSS-2 to Δ_late was decomposed into three levels, the main conclusion was fixed at the between-person level: after covariates were included, β was −0.099, with a 95% CI of [−0.211, 0.012] and *p* = 0.077, which was not significant, so H3 was not supported. The zero-order flow–pacing correlations at the two levels were *r* = −0.267 at the participant level in the main sample (*N* = 280) and *r* = −0.129 at the between-person level in the cleaned pacing sample (*n* = 249). A significant negative association appeared at the within-person level (β = −0.284, *p* < 0.001), indicating that flow and late-race pacing covaried within persons; however, this covariation did not translate into stable transmission through individual differences, so it was not included in the conclusion for H3 and is treated only as a direction for future work. The three-level estimates are shown in Table 4.

**Table 4:** Three-level estimates of FSS-2 → Δ_late.

Level	*n*	β	95% CI	*p*	R^2^
Race (after adjusting for raw race-level values)	598	−0.216	[−0.290, −0.142]	<0.001	0.077
Within-person	598	−0.284	[−0.416, −0.153]	<0.001	0.081
Between-person (main conclusion)	249	−0.099	[−0.211, 0.012]	0.077	0.095

None of the effects of the nine dimensions on Δ_late was significant after Holm correction (smallest Holm *p* = 0.208); before correction, transformation of time (*p* = 0.052) and loss of self-consciousness (*p* = 0.023) were the strongest, but the expected directional split between compatible and conflicting dimensions did not appear, and this is listed as a direction for future attention. The effects of the nine dimensions are shown in Table 5.

**Table 5:** Effects of the nine FSS-2 dimensions on Δ_late (Holm correction).

FSS-2 dimension	Zero-order *r*	Adjusted β	95% CI	*p*	Holm *p*
Challenge–skill balance	−0.104	−0.071	[−0.204, 0.062]	0.269	0.843
Action–awareness merging	−0.096	−0.072	[−0.175, 0.031]	0.154	0.843
Clear goals	−0.104	−0.071	[−0.169, 0.027]	0.140	0.843
Unambiguous feedback	−0.132	−0.104	[−0.234, 0.026]	0.107	0.749
Concentration on the task	−0.081	−0.046	[−0.147, 0.056]	0.347	0.843
Sense of control	−0.068	−0.035	[−0.138, 0.068]	0.479	0.843
Loss of self-consciousness	−0.141	−0.125	[−0.231, −0.020]	0.023	0.208
Transformation of time	−0.180	−0.156	[−0.313, 0.001]	0.052	0.415
Autotelic experience	−0.095	−0.068	[−0.165, 0.028]	0.149	0.843

### 3.4 Robustness

The indirect effect remained stable across several checks: it was 0.128 with extended covariates (mediated proportion 44.3%) and 0.099 after participant-level exclusion (*N* = 219), with a 95% CI of [0.027, 0.186]; both CIs excluded 0. The reverse mediation test (mindfulness → flow → attentional control) was also significant, with an indirect effect of 0.138 and a 95% CI of [0.077, 0.208], numerically slightly larger than, though broadly similar in magnitude to, the forward indirect effect (0.127). This result shows that the observational design and measurement timing were not sufficient to rule out the reverse path, so the causal interpretation of the mediation was limited to temporal-order inference. The robustness checks are summarised in Table 6.

**Table 6:** Summary of robustness checks.

Check	*n*	Mediated proportion	Indirect effect	95% CI
Main model	280	0.434	0.127	[0.071, 0.192]
Extended covariates	280	0.443	0.128	[0.068, 0.195]
Participant-level exclusion	219	0.387	0.099	[0.027, 0.186]
Reverse mediation (mindfulness → flow → attentional control)	280	0.524	0.138	[0.077, 0.208]

The reverse mediation was also significant, so the text acknowledges that the observational design cannot establish causal direction.

## 4 Discussion

### 4.1 Main finding: attentional control is a psychological pathway between mindfulness and race flow

In a multi-wave prospective design with competitive swimmers, attentional control mediated the association between trait mindfulness and race flow. After baseline attentional control, competitive anxiety, and competitive level were controlled, the indirect effect was 0.127 (95% CI [0.071, 0.192]), the mediated proportion was 43.4%, and the direct effect remained significant, indicating partial mediation, with the path from attentional control to flow being the strongest link in the chain. This suggests that attentional control is a core psychological pathway through which trait mindfulness is translated into race flow. Understanding flow as a cross-situational psychological experience, rather than as a mechanism specific to competitive results (Tse et al., 2022), helps to explain why this pathway appears first at the level of subjective experience. Partial mediation also means that attentional control does not exhaust the whole pathway; trait mindfulness may also act on flow through emotion regulation, motivation, or training-related psychological factors. The relationships among training stress, perfectionism, and psychological and performance indicators are themselves complex (Květon et al., 2021), which suggests that a single mediator cannot capture the entire mechanism.

### 4.2 Boundary: no sufficient evidence that the pathway extends to objective pacing

At the between-person level, race flow did not significantly predict late-race pacing deviation (β = −0.099, 95% CI [−0.211, 0.012]), so H3 was not supported; the effects of the nine flow dimensions on pacing were also all non-significant after Holm correction. This study did not find sufficient evidence for an association between race flow and objective late-race pacing at the level of stable individual differences; this absence of a detectable association should not be read as evidence that flow has no bearing on pacing. It is also consistent with the view of flow as a single, unified core experience rather than as several components that each predict behaviour independently (Tse et al., 2022). Mechanistically, accurate late-race pacing depends on continuous, controlled regulation of speed and effort, whereas objective performance is more likely to be jointly determined by factors such as pre-race routines and self-regulation strategies, rather than shaped by flow alone (Rupprecht et al., 2021). Given the additional complex effects of factors such as training load and perfectionism (Květon et al., 2021), the decoupling of subjective flow from objective pacing at the level of stable individual differences is not surprising. It should be emphasised that the within-person flow–pacing covariation observed earlier is, under this study’s framework, treated only as a direction for future work; it does not constitute evidence of transmission at the level of individual differences and does not change the main conclusion that no sufficient evidence was found for extension to objective pacing.

### 4.3 Causal interpretation: temporal order supports the direction but cannot establish causality

Although the measurement timing of the three variables (trait mindfulness at T1, attentional control at T2, and race flow post-race) was consistent with the hypothesised direction, the reverse mediation test (mindfulness → flow → attentional control) was also significant, with an indirect effect (0.138) numerically slightly larger than, though broadly similar in magnitude to, the forward path (0.127). This shows that the observational design and measurement timing were not sufficient to establish causal direction. The forward direction is nonetheless the more defensible reading on theoretical grounds. Trait mindfulness is a stable disposition assessed before competition, whereas race flow is a state arising during the event, so a durable trait is more plausibly a source of a momentary state than the reverse. Attentional control also sits closer, in established accounts of how mindfulness operates, to the regulatory mechanisms through which mindfulness shapes experience than to an outcome of that experience. Temporal precedence is therefore treated here as a supporting condition for a theoretically motivated direction rather than as its sole justification. What an observational mediation analysis can support is an association estimate corresponding to the theoretical target quantity, not a causal claim; clearly defining the estimand to be estimated, and avoiding over-causalising it, is a precondition for interpreting such results (Lundberg et al., 2021). The mediation result in this study should therefore be understood strictly as a temporal-order inference, and its causal direction awaits further testing with experimental or intervention designs.

### 4.4 Practical implications: targeting psychological experience rather than directly promising results

In practical terms, this study suggests that mindfulness training aimed at improving attentional control may help to enhance athletes’ race flow experience. However, its value is better placed within a framework of promoting athletes’ mental health and positive experience, rather than of directly promising improved competitive results (Purcell et al., 2022), which is consistent with this study’s finding that the psychological pathway was not shown to extend to objective pacing. Mindfulness programmes are feasible as a tool for improving athletes’ psychological experience, but their effects still require rigorous validation in larger samples (Evers et al., 2021). At the same time, elite athletes are not defined solely by a performance dimension; they present diverse profiles between psychological flourishing and languishing (Kuettel et al., 2021), and their athletic identity and performance pressure continually shape their psychological experience (Edison et al., 2021). It is therefore more prudent to embed attention and flow training within a more comprehensive psychological-support system. It should also be noted that, in practice, such support is affected by stigma, selection pressure, and barriers to help-seeking (Cosh et al., 2024), and intervention designs should take these real-world constraints into account.

### 4.5 Limitations

This study has several limitations. The design was observational, the reverse path could not be ruled out, and causal direction could not be established. The test–retest correlation of attentional control was only *r* = 0.35, indicating a substantial occasion-specific component, so ACS_T2 is best read as near-race attentional control rather than a stable trait, and future work would benefit from combining self-report with behavioural or objective measures of attention; race flow was self-reported after the race, and result information was immediately visible (results were known in about 35% of races), which raises a risk of halo contamination. The AVE based on a principal-component approximation was below 0.50 for all constructs, including the comparatively unidimensional attentional-control scale; because a principal-component approximation does not separate common from unique variance and tends to inflate rather than deflate loadings, this shortfall is read as a genuine, conservative result rather than as an artefact of underestimation. A full latent-variable confirmatory factor model was not fitted: the analysis was specified on composite (person-mean) indicators by design, and simultaneously modelling the item-level structure of several long scales—most notably the 39-item FFMQ and the 36-item FSS-2—would have required estimating a large number of parameters relative to the available sample (*N* = 280), with an unfavourable parameter-to-observation ratio and a corresponding risk of unstable or non-converging solutions. Because AVE is a conservative index, convergent validity therefore rested on the established construct validity and high internal consistency of each scale (α = 0.80–0.95), which can indicate adequate convergent validity even when AVE falls below 0.50 (Fornell and Larcker, 1981), rather than on sample-specific AVE; discriminant validity was supported by HTMT. Inferences at the latent-variable level must nonetheless remain cautious and would be firmer under confirmatory factor or latent-variable modelling—if necessary with item parcelling—in a larger sample; the study was also not preregistered, so confirmatory inference cannot be independently verified. Contextual variables not included in the model—such as social support and athletic identity—may affect both pre-race and post-race psychological states (Hagiwara et al., 2021), and competitive, academic, or training load together with mental health jointly shape athletes’ competitive experience, which suggests that more contextual variables should be controlled (Kegelaers et al., 2022). In addition, the sample was limited to competitive swimmers of a single event and a single competitive level, so generalising results based on a specific population and context requires particular caution (Yarkoni, 2022).

### 4.6 Future directions

Future research can extend this work in several directions. Extending individual-level flow to the team or training-group level, and examining the formation of group flow with physiological indicators such as cardiovascular synchronisation, is a route worth pursuing (Snijdewint and Scheepers, 2023); the within-race flow–pacing state covariation observed here also deserves further characterisation within a within-race dynamic framework. Another route is to track flow fluctuations in real time using behavioural data from continuous tasks, so as to reduce the bias introduced by a single post-race self-report (Oliveira et al., 2021) and to test the dynamic relationship between flow and pacing at finer temporal resolution. On this basis, using a randomised intervention design to manipulate attentional control would directly test the temporal pathway proposed here and clarify, at the causal level, the role of attentional control as the pathway linking mindfulness to race flow.

## 5 Conclusion

In a multi-wave prospective observational design with competitive swimmers, this study examined the relationships among trait mindfulness, attentional control, and race flow, and treated objective pacing accuracy as an exploratory downstream outcome. The results show that attentional control is a psychological pathway between trait mindfulness and race flow: after baseline attentional control, competitive anxiety, and competitive level were controlled, trait mindfulness was linked to race flow through near-race attentional control, indicating partial mediation, with the path from attentional control to flow being the strongest link in the chain. This pathway provides a testable psychological mechanism for understanding how mindfulness is translated into flow under real competition, and it suggests that attentional regulation may be a key intermediate link through which mindfulness works in competitive settings. It should be emphasised that, because the design was observational and the measurement timing was not sufficient to rule out the reverse path, these relationships should be understood as temporal-order inferences rather than causal conclusions.

At the same time, this study clarified the boundary of this psychological pathway. At the level of stable individual differences, race flow was not significantly associated with objective late-race pacing accuracy, and the effects of the nine flow dimensions on pacing were all non-significant after correction for multiple comparisons, so this study did not find sufficient evidence that the pathway extends to objective performance at the level of stable individual differences. This boundary suggests that the value of mindfulness training aimed at improving attentional control and race flow is better positioned as improving athletes’ competitive psychological experience, rather than as directly promising gains in objective results. Overall, this study characterises attentional control as a psychological pathway linking mindfulness to race flow and clarifies the scope within which this pathway applies, laying a foundation for subsequent studies to test its causal direction with randomised intervention designs and to further examine the relationship between flow and objective performance.

## Data Availability

The raw data supporting the conclusions of this article will be made available by the authors, without undue reservation.

## References

[ref1] Ashinoff B. K. Abu-Akel A. (2021). Hyperfocus: the forgotten frontier of attention. Psychol. Res. 85, 1–19. doi: 10.1007/s00426-019-01245-8, 31541305 PMC7851038

[ref2] Baminiwatta A. Solangaarachchi I. (2021). Trends and developments in mindfulness research over 55 years: a bibliometric analysis of publications indexed in web of science. Mindfulness 12, 2099–2116. doi: 10.1007/s12671-021-01681-x, 34306245 PMC8282773

[ref3] Cosh S. McNeil D. Jefferys A. Clark L. Tully P. J. (2024). Athlete mental health help-seeking: a systematic review and meta-analysis of rates, barriers and facilitators. Psychol. Sport Exerc. 71:102586. doi: 10.1016/j.psychsport.2023.102586 38128709

[ref4] Domínguez-González J. A. Reigal R. E. Morales-Sánchez V. Hernández-Mendo A. (2024). Analysis of the sports psychological profile, competitive anxiety, self-confidence and flow state in young football players. Sports 12:18. doi: 10.3390/sports12010018 PMC10818857 38251294

[ref5] Edison B. R. Christino M. A. Rizzone K. H. (2021). Athletic identity in youth athletes: a systematic review of the literature. Int. J. Environ. Res. Public Health 18:7331. doi: 10.3390/ijerph18147331, 34299786 PMC8305814

[ref6] Evers A. Klatt M. Carvalho J. (2021). The adaptation and evaluation of a pilot mindfulness intervention promoting mental health in student athletes. J. Clin. Sport Psychol. 15, 1–21. doi: 10.1123/jcsp.2019-0083

[ref7] Fornell C. Larcker D. F. (1981). Evaluating structural equation models with unobservable variables and measurement error. J. Mark. Res. 18, 39–50. doi: 10.1177/002224378101800104

[ref8] Goddard S. G. Stevens C. J. Jackman P. C. Swann C. (2023). A systematic review of flow interventions in sport and exercise. Int. Rev. Sport Exerc. Psychol. 16, 657–692. doi: 10.1080/1750984X.2021.1923055

[ref9] Goldberg S. B. Riordan K. M. Sun S. Davidson R. J. (2022). The empirical status of mindfulness-based interventions: a systematic review of 44 meta-analyses of randomized controlled trials. Perspect. Psychol. Sci. 17, 108–130. doi: 10.1177/1745691620968771, 33593124 PMC8364929

[ref10] Hagiwara G. Tsunokawa T. Iwatsuki T. Shimozono H. Kawazura T. (2021). Relationships among student-athletes' identity, mental health, and social support in Japanese student-athletes during the COVID-19 pandemic. Int. J. Environ. Res. Public Health 18:7032. doi: 10.3390/ijerph18137032, 34209463 PMC8297159

[ref11] Kegelaers J. Wylleman P. Defruyt S. Praet L. Stambulova N. Torregrossa M. . (2022). The mental health of student-athletes: a systematic scoping review. Int. Rev. Sport Exerc. Psychol. 17, 848–881. doi: 10.1080/1750984X.2022.2095657

[ref12] Kuettel A. Pedersen A. K. Larsen C. H. (2021). To flourish or languish, that is the question: exploring the mental health profiles of Danish elite athletes. Psychol. Sport Exerc. 52:101837. doi: 10.1016/j.psychsport.2020.101837

[ref13] Květon P. Jelínek M. Burešová I. (2021). The role of perfectionism in predicting athlete burnout, training distress, and sports performance: a short-term and long-term longitudinal perspective. J. Sports Sci. 39, 1969–1979. doi: 10.1080/02640414.2021.1912451, 33818303

[ref14] Lochbaum M. Stoner E. Hefner T. Cooper S. Lane A. M. (2022). Sport psychology and performance meta-analyses: a systematic review of the literature. PLoS One 17:e0263408. doi: 10.1371/journal.pone.0263408, 35171944 PMC8849618

[ref15] Lüdecke D. Ben-Shachar M. S. Patil I. Waggoner P. Makowski D. (2021). Performance: an R package for assessment, comparison and testing of statistical models. J. Open Source Softw. 6:3139. doi: 10.21105/joss.03139

[ref16] Lundberg I. Johnson R. Stewart B. M. (2021). What is your estimand? Defining the target quantity connects statistical evidence to theory. Am. Sociol. Rev. 86, 532–565. doi: 10.1177/00031224211004187

[ref17] Norsworthy C. Dimmock J. A. Nicholas J. Krause A. Jackson B. (2023a). Psychological flow training: feasibility and preliminary efficacy of an educational intervention on flow. Int. J. Appl. Posit. Psychol. 8, 655–684. doi: 10.1007/s41042-023-00105-7 PMC10204032 37361627

[ref18] Norsworthy C. Jackson B. Dimmock J. A. (2021). Advancing our understanding of psychological flow: a scoping review of conceptualizations, measurements, and applications. Psychol. Bull. 147, 806–827. doi: 10.1037/bul0000337 34898235

[ref19] Norsworthy C. Jackson B. Dimmock J. A. (2023b). Psychological flow scale (PFS): development and preliminary validation of a new flow instrument that measures the core experience of flow to reflect recent conceptual advancements. Int. J. Appl. Posit. Psychol. 8, 309–337. doi: 10.1007/s41042-023-00092-9

[ref20] Oliveira W. Tenório K. Hamari J. Pastushenko O. Isotani S. (2021). Predicting students' flow experience through behavior data in gamified educational systems. Smart Learn. Environ. 8:30. doi: 10.1186/s40561-021-00175-6

[ref21] Peifer C. Wolters G. Harmat L. Heutte J. Tan J. Freire T. . (2022). A scoping review of flow research. Front. Psychol. 12:815665. doi: 10.3389/fpsyg.2021.815665 PMC9022035 35465560

[ref22] Purcell R. Pilkington V. Carberry S. Reid D. Gwyther K. Hall K. . (2022). An evidence-informed framework to promote mental wellbeing in elite sport. Front. Psychol. 13:780359. doi: 10.3389/fpsyg.2022.780359, 35250720 PMC8890033

[ref23] Rupprecht A. G. O. Tran U. S. Gröpel P. (2021). The effectiveness of pre-performance routines in sports: a meta-analysis. Int. Rev. Sport Exerc. Psychol. 17, 1–26. doi: 10.1080/1750984X.2020.1859322

[ref24] Scheepers D. Keller J. (2022). On the physiology of flow: bridging flow theory with the biopsychosocial model of challenge and threat. Int. J. Psychophysiol. 180, 10–16. doi: 10.1016/j.ijpsycho.2022.07.004, 36273715

[ref25] Snijdewint J. A. Scheepers D. (2023). Group-based flow: the influence of cardiovascular synchronization and identifiability. Psychophysiology 60:e14233. doi: 10.1111/psyp.14233 36458474

[ref26] Tse D. C. K. Nakamura J. Csikszentmihalyi M. (2022). Flow experiences across adulthood: preliminary findings on the continuity hypothesis. J. Happiness Stud. 23, 2517–2540. doi: 10.1007/s10902-022-00519-2

[ref27] van der Linden D. Tops M. Bakker A. B. (2021). The neuroscience of the flow state: involvement of the locus coeruleus norepinephrine system. Front. Psychol. 12:645498. doi: 10.3389/fpsyg.2021.645498, 33935902 PMC8079660

[ref28] van Lissa C. J. (2022). tidySEM: tidy structural equation modeling. J. Open Source Softw. 7:4295. doi: 10.21105/joss.04295

[ref29] Wang Y. Lei S. M. Fan J. (2023). Effects of mindfulness-based interventions on promoting athletic performance and related factors among athletes: a systematic review and meta-analysis of randomized controlled trial. Int. J. Environ. Res. Public Health 20:2038. doi: 10.3390/ijerph20032038, 36767403 PMC9915077

[ref30] Weder B. J. (2022). Mindfulness in the focus of the neurosciences: the contribution of neuroimaging to the understanding of mindfulness. Front. Behav. Neurosci. 16:928522. doi: 10.3389/fnbeh.2022.928522, 36325155 PMC9622333

[ref31] Yarkoni T. (2022). The generalizability crisis. Behav. Brain Sci. 45:e1. doi: 10.1017/S0140525X20001685, 33342451 PMC10681374

[ref32] Zhang D. Lee E. K. P. Mak E. C. W. Ho C. Y. Wong S. Y. S. (2021). Mindfulness-based interventions: an overall review. Br. Med. Bull. 138, 41–57. doi: 10.1093/bmb/ldab005, 33884400 PMC8083197

